# Beyond the 9 to 5: A Cross-sectional Survey of Adult Antimicrobial Stewardship Programs in the United States on Their Initiatives and Resources Based on On-call Model Participation

**DOI:** 10.1093/ofid/ofaf722

**Published:** 2025-12-15

**Authors:** Samantha Brace, Gustavo Rey Alvira-Arill, Aaron Hamby, Rachel Burgoon, Zachary Gruss, Taylor Morrisette, Alexandra Mills, Richard Lueking, Stephen Thacker, Krutika Mediwala Hornback

**Affiliations:** Department of Pharmacy Services, Medical University of South Carolina Health, Charleston, South Carolina, USA; Department of Pharmacy Services, Medical University of South Carolina Health, Charleston, South Carolina, USA; Department of Clinical Pharmacy and Outcomes Sciences, Medical University of South Carolina College of Pharmacy, Charleston, South Carolina, USA; Department of Pharmacy Services, Medical University of South Carolina Health, Charleston, South Carolina, USA; Department of Pharmacy Services, Medical University of South Carolina Health, Charleston, South Carolina, USA; Department of Pharmacy Services, Medical University of South Carolina Health, Charleston, South Carolina, USA; Department of Pharmacy Services, Medical University of South Carolina Health, Charleston, South Carolina, USA; Department of Clinical Pharmacy and Outcomes Sciences, Medical University of South Carolina College of Pharmacy, Charleston, South Carolina, USA; Division of Infectious Diseases, Department of Internal Medicine, Medical University of South Carolina Health, Charleston, South Carolina, USA; Division of Infectious Diseases, Department of Internal Medicine, Medical University of South Carolina Health, Charleston, South Carolina, USA; Division of Infectious Diseases, Department of Pediatrics, Medical University of South Carolina Health, Charleston, South Carolina, USA; Department of Pharmacy Services, Medical University of South Carolina Health, Charleston, South Carolina, USA

**Keywords:** antimicrobial stewardship, cross-sectional studies, healthcare surveys, personnel staffing and scheduling

## Abstract

**Background:**

Antimicrobial stewardship programs (ASPs) aim to optimize antimicrobial use through coordinated interventions that improve patient outcomes and reduce adverse events. While guidance exists from organizations including the Centers for Disease Control and Prevention and the Infectious Diseases Society of America, recommendations on effort allocation, working hours, and initiatives remain unclear.

**Methods:**

This cross-sectional survey assessed the institutional structure, effort allocation, initiatives, and on-call participation of adult ASPs in the United States from September to October 2024. The survey was distributed via email to several ASP-related listservs. Respondents indicating on-call participation were also inquired about working hours, initiatives performed, participants, and compensation.

**Results:**

Of 69 responses, most were from academic medical centers (59%) or community hospitals (35%), with 65% covering >500 beds. ASPs were often system-wide (78%) and primarily funded by their respective departments of pharmacy (87%). Common initiatives performed by all ASPs include answering ASP/infectious diseases questions, therapy de-escalation, and prospective audit and feedback. Twenty-four (69%) respondents indicated having an on-call model, with said programs reporting higher median inpatient full-time equivalents (FTEs) for physicians (0.5 vs 0.25) and pharmacists (2.9 vs 1.45) than those without. Commonly performed after-hours initiatives include preauthorization and answering microbiology inquiries. On-call coverage was generally performed during weekend daytimes and holidays, most often by pharmacists.

**Conclusions:**

This survey highlights differences in structure, effort allocation, and initiatives of ASPs based on on-call participation. Institutions participating in on-call reported higher FTE assignments for physicians and pharmacists and were more likely to perform time-sensitive initiatives.

## Teaser

The survey assesses US antimicrobial stewardship programs and highlights differences in institutional structure, effort allocation, and initiatives performed based on on-call participation. Findings suggest higher full-time equivalent assignments and more frequent time-sensitive initiatives with an on-call model.

Antimicrobial stewardship programs (ASPs) aim to improve and measure appropriate antimicrobial use through coordinated interventions, which result in improved patient outcomes and reduced adverse events [1, 2]. Since 2017, implementation of these programs has been a requirement for United States (US) hospital accreditation, with core members and priority interventions specified by several organizations including the Centers for Disease Control and Prevention (CDC) and the Infectious Diseases Society of America (IDSA) [3–8]. ASPs should be supported by a multidisciplinary team inclusive of physicians, pharmacists, clinical microbiologists, infection preventionists, information technology specialists, and nurses to optimize antimicrobial use through prospective audit and feedback (PAF), preauthorization (PRA), guideline implementation, and other initiatives [2–4]. Despite available guidance and mandates for ASP implementation, recommendations for optimal participants and associated effort allocation, working hours, and specific initiatives remain unclear.

Reports of ASP participants and associated effort allocation vary by institution type and initiatives performed [9–16]. In the US, median full-time equivalents (FTEs) assigned range from 0.24 to 0.46 for physicians and 0.61 to 1.5 for pharmacists, which was generally dedicated toward inpatient ASP initiatives including PRA and PAF [9–13]. While other members are reportedly involved with ASP initiatives, such as infection preventionists and information technology specialists, FTE assignments are infrequently reported. Reports from France and the United Kingdom indicate comparatively higher FTE assignments for physicians and pharmacists, but this varied based on local standards for different institution sizes [14–16]. Regardless, initiatives performed were similarly described with a focus on review of inpatient antimicrobial utilization. These reports provide insight into the effort allocation required for ASP activities but working hours for these services are neither routinely reported on nor mentioned in available guidance.

Generally, ASP initiatives are performed during traditional working hours; however, some warrant greater availability of ASP staff for PRA of restricted antimicrobials [1–5]. In addition, reports of PAF and sterile-site culture review performed during weekend hours suggest potential benefits including reductions in antimicrobial utilization, time to targeted therapy, length of stay, and in-hospital mortality [17–22]. While ASP initiatives performed after hours may improve patient outcomes, compensation and coordination of staff for these activities have not been described. Of aforementioned reports, initiatives performed during weekend hours often featured pharmacists and pharmacy residents for provision of PRA and PAF, and infectious diseases (ID)–trained physicians for sterile-site culture review [17–22]. Therefore, the objective of this survey was to characterize the structure, initiatives, and working hours of ASPs while distinguishing programs that report after-hours activity with an on-call model.

## METHODS

### General Study Design

This was a cross-sectional, investigator-developed survey designed to assess institutional structure, effort allocation, initiatives, and on-call participation for ASPs in the US from September to October 2024. The survey was distributed by email to the American College of Clinical Pharmacy (ACCP) Infectious Diseases Practice and Research Network, IDSA, Infectious Diseases Educator Network, Pediatric Infectious Diseases Society, and Vizient Antimicrobial Stewardship listservs. In addition, one reminder email was sent approximately halfway through the open survey period. Survey recipients were asked to have one individual from their institution respond, and completed surveys were included for assessment. If multiple responses were received from an institution, then the most complete response was included. For this report, responses indicating the institution type as a pediatric community hospital or pediatric academic medical center were excluded.

### Survey Development

Survey items were developed from a multidisciplinary group of ID and antimicrobial stewardship pharmacists and physicians. The survey was modeled from a previous informal ACCP intake survey assessing initiatives and personnel of ASPs with an on-call model. From this, the investigator group created a formal survey with 63 potential questions designed to assess 3 sections (Supplementary material). The first section inquired on institutional demographic information including geographic location, institution type, and approximate number of patients covered by the associated ASP. The second section inquired on ASP structure including participants and associated FTE assignment, funding and reporting hierarchy, and inclusion of ambulatory or outpatient services. The third section inquired about initiatives taken by the respondent institution including PRA, PAF, and sterile-site culture review. In addition, communication and documentation of interventions performed were also assessed.

For the purposes of this survey, an on-call model was defined by an ASP conducting initiatives outside of traditional working hours (for ex: Monday through Friday, 8 Am to 5 Pm). This included coverage during weekday evenings (for ex: Monday through Friday, 5 Pm to 8 Am), weekend daytime hours (for ex: Saturday through Sunday, 8 Am to 5 Pm), weekend evenings (for ex: Saturday through Sunday, 5 Pm to 8 Am), and major US holidays (eg, Christmas, Thanksgiving, Independence Day). The timing for on-call transitions depended on the respondent's interpretation but was indicated by confirming coverage during those periods. Respondents who indicated that their ASP participated in an on-call model were also inquired about initiatives performed outside of traditional working hours, working hours for on-call, participants of the model, and compensation. Last, respondents were inquired about shifts when the on-call model was active and the number of shifts that were assigned to participating members, which were defined as weekday evenings/nights, weekend daytimes, weekend evenings/nights, and major work holidays.

Respondents were required to answer each survey item before proceeding to the next section, and branching logic was utilized based on answers entered on pertinent questions. Free-text comments were enabled for each question, and a final comment was included to elaborate on any other items of importance. Following survey build, pilot testing was performed by a subset of the study investigators to assess interpretability, identify errors, and estimate time to completion. The study protocol was reviewed by the Institutional Review Board of the Medical University of South Carolina (MUSC) and determined to be exempt from human research subject regulations. The survey was created and distributed electronically using the Research Electronic Data Capture (REDCap) application hosted at MUSC [23, 24].

### Data Analysis

**Figure 1:**
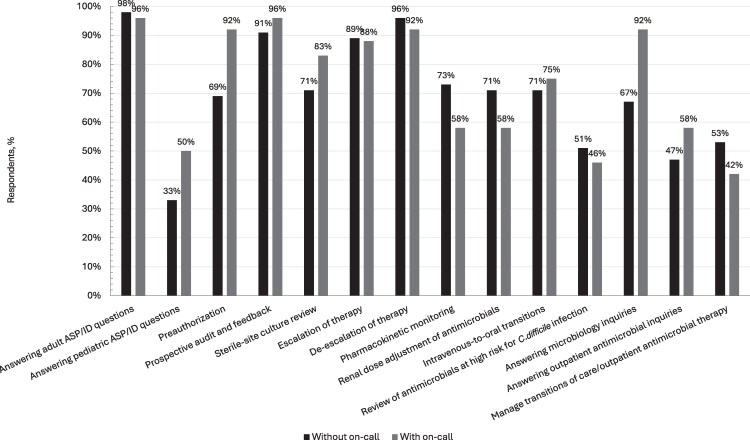
Antimicrobial stewardship program initiatives based on on-call participation. With on-call, n = 24; Without on-call, n = 45. Abbreviations: ASP, antimicrobial stewardship program; *C difficile*, *Clostridioides difficile*; ID, infectious diseases.

**Figure 2.**
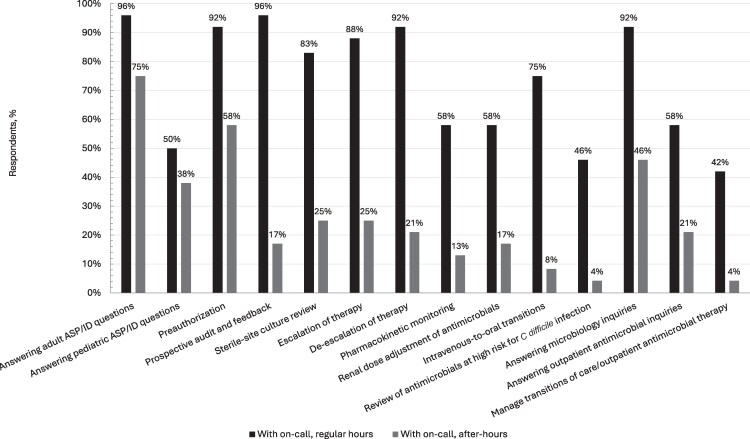
Antimicrobial stewardship program initiatives for on-call programs based on timing. With on-call, n = 24. Abbreviations: ASP, antimicrobial stewardship program; *C difficile*, *Clostridioides difficile*; ID, infectious diseases.

Proportions were used to describe categorical variables, and medians with interquartile range (IQR) were used for continuous variables. Free-text comments and responses were abstracted and grouped based on similar concepts or phrasing. Program characteristics were delineated by on-call participation, but no statistical analysis was planned or performed. This decision was based on the exploratory nature of this study, which precluded meaningful statistical inference. Data processing and wrangling were performed using R software version 4.4.2 (R Foundation for Statistical Computing, Vienna, Austria) and graphical visualization was performed using Excel software version 2502 (Microsoft Corporation, Redmond, Washington). For visualization of differences in initiatives performed, Figure 1 illustrates differences based on on-call model participation. Figure 2 illustrates differences in initiatives performed based on working hours for on-call programs.

## RESULTS

The survey was completed in 95 of 141 responses, with 91 complete responses assessed after removing duplicates. Of the 91 responses assessed, 22 responses indicated the institution as a pediatric community hospital or pediatric academic medical center, so 69 responses were included for review. Responses were received from all geographical regions of the US, with most received from the Northeast and Midwest. Most respondents indicated that their institution was either an academic medical center or a community hospital, with most programs covering >500 beds (45/69 [65%]). Of the survey responses included, 24 of 69 (35%) indicated that their institution participated in an on-call model. Summary statistics of demographic information and ASP structure from respondents are delineated by aggregate and based on on-call participation in Table 1.

**Table 1.** Demographic and Structural Characteristics of Antimicrobial Stewardship Programs

Characteristic	Responses, No. (%)		
	With On-call (n = 24)	Without On-call (n = 45)	Overall (n = 69)
Geographical location			
Midwest	6 (25)	11 (24)	17 (25)
Northeast	8 (33)	11 (24)	19 (28)
Southeast	6 (25)	8 (18)	14 (20)
Southwest	3 (13)	3 (7)	6 (8)
West	1 (4)	12 (27)	13 (19)
Institution type			
Academic medical center	19 (79)	22 (49)	41 (59)
Community hospital	5 (21)	19 (42)	24 (35)
Critical access hospital	0 (0)	1 (2.25)	1 (1.5)
Specialty hospital	0 (0)	1 (2.25)	1 (1.5)
Federal facility	0 (0)	1 (2.25)	1 (1.5)
Long-term acute care facility	0 (0)	1 (2.25)	1 (1.5)
Institution size			
<100	0 (0)	2 (5)	2 (3)
101–250	1 (4)	5 (11)	6 (9)
251–500	5 (21)	11 (24)	16 (23)
>500	18 (75)	27 (60)	45 (65)
ASP organization			
System-wide, with flagship hospital	10 (42)	10 (22)	20 (29)
System-wide, hospitals act independently	10 (42)	24 (53)	34 (49)
Single facility	4 (16)	11 (25)	15 (22)
Presence of ambulatory or outpatient ASP	5 (21)	14 (31)	19 (28)
Presence of OPAT/COpAT program	11 (46)	22 (49)	33 (48)
ASP reporting department(s)^a^			
Quality and Safety	6 (25)	10 (22)	16 (23)
Medicine	1 (4.2)	5 (11)	6 (8.7)
Pharmacy	4 (17)	15 (33)	19 (28)
Report to respective department	13 (54)	15 (33)	28 (41)
Funding source(s)^a^			
Quality and Safety	2 (8.3)	6 (13)	8 (12)
Medicine	4 (17)	14 (31)	18 (26)
Pharmacy	21 (88)	39 (87)	60 (87)
No funding	1 (4.2)	2 (4.4)	3 (4)
Participating members^a^			
Physician(s)	24 (100)	42 (93)	66 (96)
Pharmacist(s)	24 (100)	44 (98)	68 (99)
Clinical microbiologist(s)	12 (50)	27 (60)	39 (57)
Information analyst(s)	10 (42)	15 (33)	25 (36)
Infection prevention(s)	12 (50)	28 (62)	40 (58)
Hospital epidemiologist(s)	10 (42)	9 (20)	19 (28)
Physician leadership for ASP			
No physician leadership	0 (0)	3 (6)	3 (4)
One physician leader	15 (63)	34 (76)	49 (71)
Multiple physician leaders	9 (37)	8 (18)	17 (25)
Pharmacist leadership for ASP			
No pharmacist leadership	5 (20)	2 (5)	7 (10)
One pharmacist leader	16 (67)	32 (71)	48 (70)
Multiple pharmacist leaders	3 (13)	11 (24)	14 (20)

Abbreviations: ASP, antimicrobial stewardship program; COpAT, complex outpatient antimicrobial therapy; OPAT, outpatient parenteral antimicrobial therapy.

^a^Respondents could have selected >1 category.

Table 1 also delineates structural characteristics of ASPs, with or without on-call participation, including participants and hierarchy for funding and reporting by aggregate. Most respondents indicated system-wide organization with a flagship hospital or independently acting hospitals. Funding and reporting structures varied, with most ASPs reporting to and funded by their pharmacy departments. Almost all respondents indicated physicians and pharmacists participating in ASP, with varied participation of other specialists including infection preventionists, clinical microbiologists, information analysts, and hospital epidemiologists. ASP leadership of respondents was mostly inclusive of one physician and one pharmacist, but a higher proportion of respondents with an on-call ASP model indicated lack of pharmacist leadership compared to those without.

ASP inpatient and outpatient effort reported by respondents based on on-call participation are outlined in Table 2. Median inpatient FTE assignments for ASP physicians and pharmacists were higher for programs participating in an on-call model. FTE assignments increased with institution size as median (IQR) physician FTEs were 0.18 (0.10–0.25) for hospitals with <100 beds, 0.25 (0.20–0.25) for 101–250 beds, 0.27 (0.10–0.50) for 251–500 beds, and 0.50 (0.25–1.00) for >500 beds. For pharmacists, median (IQR) FTEs were 1.00 for <100 beds, 0.90 (0.80–0.95) for 101–250 beds, 0.75 (0.50–1.00) for 251–500 beds, and 2.25 (2.00–3.00) for >500 beds. The proportion of respondents indicating outpatient ASP effort for physicians and pharmacists was comparatively lower than inpatient. Respondents indicated that pharmacists supported the ID consult service by direct rounding (31/69 [45%]) or answering questions (19/69 [28%]), which was similar between those who do and do not participate in an on-call model. Effort allocation from other specialists was variably indicated by respondents, but median FTE assignments were comparatively lower to pharmacists and physicians regardless of location or on-call participation. In addition, the number of other specialists with allocated effort and median FTE reported was lower for those with an on-call program compared to those without.

**Table 2.** Antimicrobial Stewardship Program Participant Effort and Assigned Full-Time Equivalent

Participants	Responses, No. (%); FTE, Median (IQR)		
	With On-call (n = 24)	Without On-call (n = 45)	Overall (n = 69)
Inpatient			
Physicians	19 (79); 0.50 (0.40–1.00)	30 (67); 0.25 (0.13–0.60)	49 (71); 0.45 (0.20–0.75)
Pharmacists	20 (83); 2.90 (1.00–3.35)	36 (80); 1.45 (0.90–2.00)	56 (81); 2.00 (1.00–3.00)
Clinical microbiologists	1 (4.2); 0.20 (NA)	9 (20); 0.80 (0.20–1.00)	10 (14); 0.65 (0.20–1.00)
Information specialists	2 (8.3); 0.20 (NA)	4 (8.9); 0.38 (0.18–0.50)	6 (8.7); 0.23 (0.20–0.50)
Infection preventionists	2 (8.3); 0.75 (0.50–1.00)	9 (20); 1.00 (0.10–1.00)	11 (16); 1.00 (0.10–1.00)
Hospital epidemiologists	2 (8.3); 0.38 (0.25–0.50)	2 (4.4); 0.63 (0.25–1.00)	4 (5.8); 0.38 (0.25–0.75)
Outpatient			
Physicians	3 (13); 0.25 (0.10–0.30)	2 (4.4); 1.13 (0.25–2.00)	5 (7.2); 0.25 (0.25–0.30)
Pharmacists	4 (17); 1.00 (0.80–1.00)	7 (16); 1.00 (0.50–1.00)	11 (16); 1.00 (0.50–1.00)
Clinical microbiologists	0 (0); NA	3 (6.7); 0.50 (0.20–1.00)	3 (4.3); 0.50 (0.20–1.00)
Information specialists	1 (4.2); 0.05 (NA)	2 (4.4); 0.30 (0.10–0.50)	3 (4.3); 0.10 (0.05–0.50)
Infection preventionists	0 (0); NA	0 (0); NA	0 (0); NA
Hospital epidemiologists	0 (0); NA	0 (0); NA	0 (0); NA

Abbreviations: FTE, full-time equivalent; IQR, interquartile range; NA, not applicable.

Initiatives performed during regular working hours for ASPs reported by respondents based on on-call participation are displayed in Figure 1. Regardless of on-call participation, common initiatives include answering adult ASP/ID questions (67/69 [97%]), de-escalation of therapy (65/69 [94%]), PAF (64/69 [93%]), and escalation of therapy (61/69 [88%]). Conversely, PRA, answering microbiology inquiries, pharmacokinetic monitoring, and sterile-site culture review appeared to differ based on on-call participation. Initiatives less frequently performed include review of antimicrobials at high risk for *Clostridioides difficile* infection (34/69 [49%]), managing transitions of care or outpatient antimicrobial therapy (34/69 [49%]), and answering pediatric ASP/ID questions (27/69 [39%]). Documentation and communication methods varied widely, with most programs using electronic health record–integrated tools. Of all respondents, only one ASP without an on-call model indicated billing for their services.

On-call coverage was most common during weekends and holidays, primarily staffed by pharmacists (15/24 [63%]) and often conducted remotely (20/24 [83%]) (Table 3). Of note, physician assignments may have overlapped with consultation coverage but could not be differentiated against based on the survey. Compared to regular working hours, respondents indicated that fewer initiatives were performed during on-call staffing with reductions in the proportion of institutions performing PAF, sterile-site culture review, and PRA (Figure 2). Only 9 of 24 (38%) respondents indicated their institution receiving some form of compensation for on-call staffing, which was commonly in the form of a post-call day off (4/24 [17%]) or incentive pay (4/24 [17%]).

**Table 3.** Structure of Antimicrobial Stewardship Programs During On-call Initiatives

Characteristic	Responses, No. (%) (n = 24)
ASP on-call availability^a^	
Weekday evening/night	17 (71)
Weekend daytime	22 (92)
Weekend evening/night	14 (58)
Major holiday(s)	20 (83)
Location for ASP on-call service	
Remote	16 (66)
On-site	4 (17)
Remote or on-site	4 (17)
Physician assignment for ASP on-call (n = 11)	
1–5 shifts per year	0 (0)
5–10 shifts per year	3 (27)
10–15 shifts per year	1 (9.1)
15–20 shifts per year	1 (9.1)
>20 shifts per year	6 (55)
Pharmacist assignment for ASP on-call (n = 15)	
1–5 shifts per year	0 (0)
5–10 shifts per year	3 (20)
10–15 shifts per year	5 (33)
15–20 shifts per year	1 (6.7)
>20 shifts per year	6 (40)
ID medical fellow assignment for ASP on-call (n = 3)	
1–5 shifts per year	0 (0)
5–10 shifts per year	1 (33)
10–15 shifts per year	0 (0)
15–20 shifts per year	0 (0)
>20 shifts per year	2 (67)
Pharmacy resident assignment for ASP on-call (n = 10)	
1–5 shifts per year	0 (0)
5–10 shifts per year	2 (20)
10–15 shifts per year	2 (20)
15–20 shifts per year	3 (30)
>20 shifts per year	3 (30)

Abbreviations: ASP, antimicrobial stewardship program; ID, infectious diseases.

^a^Respondents could have selected >1 category.

## DISCUSSION

From evidence previously reviewed, ASP initiatives performed after hours have the potential to improve patient outcomes through including reductions in antimicrobial utilization, time to targeted therapy, length of stay, and in-hospital mortality [17–22]. However, guidance on staffing and initiatives performed outside of traditional working hours are currently unavailable [1–5]. This survey delineates institutional demographics, structure, initiatives, and working hours of ASPs based on participation in an on-call model. Most respondents were from academic medical centers or community hospitals with ASP covering >500 beds, and approximately one-third of those ASP functioned through an on-call model. Respondents indicated a variety of participants in their ASP; however, most allocated effort was to physicians and pharmacists with higher FTE assignments to those participating in on-call. Comparatively lower allocated effort was reported for outpatient ASP activities, regardless of participant role. Of initiatives performed, a similar proportion of respondents indicated performing specified initiatives aside from PRA and answering microbiology inquiries, which were more frequently reported in ASPs with an on-call model. Last, respondents indicated that on-call was available during most times except weekend evenings/nights and the initiatives performed were comparatively reduced during on-call staffing.

As previously stated, there is guidance available from several organizations on ASP implementation, but no specific recommendations are provided for optimal effort allocation of team members and structure. Prior evidence assessing effort allocation of ASP members demonstrated that assigned FTEs for physicians and pharmacists increased relative to bed size [10]. Doernberg et al reported median (IQR) physician FTEs ranging from 0.27 (0–0.87) in hospitals with <100 beds to 0.46 (0.2–1.4) in hospitals with >1000 beds, while pharmacist FTEs ranged from 0.61 (0–2.0) to 1.5 (0.5–3.1) [10]. Our study had similar findings with increased median inpatient FTE assignments for physicians and pharmacists within larger institutions, alongside an increasing proportion reporting on-call participation. Outpatient FTEs were less frequently reported and largely reserved for institutions with >500 beds. For other ASP members, such as informatics and data analysts, FTE assignments varied and were inconsistently reported, regardless of bed size or on-call participation.

On structural characteristics, most respondents indicated system-wide ASP organization consistent with a smaller survey of select ASP programs, with a higher proportion of respondents indicating coordination through a flagship hospital for ASPs with an on-call model [25]. Funding was most commonly reported from their respective departments of pharmacy, even when programs reported to other departments such as quality and safety, highlighting a disconnect between reporting and financial support [26]. This mixed reporting structure is optimal for patient safety and cost avoidance but may prove difficult for billing opportunities. Most respondents indicated that their ASP was co-led by physicians and pharmacists (59/69 [86%]) in alignment with established guidelines [2, 3, 6–8]. Fee-for-service billing was uncommon, with only one respondent reporting billing for ASP services: this involved ASP physicians rounding on patients without ID consultation and being accompanied by an ASP pharmacist.

Respondents reported that common ASP initiatives performed include answering adult ASP/ID questions, de-escalation of therapy, and PAF. Respondents indicating on-call model participation were more likely to perform PRA, answer microbiology inquiries, and review positive sterile-site cultures compared to those who do not. These findings are sensible as ASPs with an on-call model would be more readily available to handle time-sensitive initiatives including PRA and positive sterile-site culture review, with benefits demonstrated by both interventions performed after hours [18, 20–22]. Conversely, programs without an on-call model more frequently reported performing pharmacokinetic monitoring and dose adjustment of antimicrobials. Although this survey did not specifically assess initiatives performed by pharmacists without formal ASP responsibilities, the observed differences may reflect prioritization of core stewardship activities—such as PAF and PRA—while general dosing and monitoring responsibilities often remain within the scope of pharmacists without formal ASP responsibilities [26–28].

These findings align with results from Doernberg et al in that most academic medical centers employed dedicated stewardship pharmacists and physicians; meanwhile, nearly one-third of smaller hospitals lacked any dedicated FTEs, potentially relying instead on infection preventionists or clinical pharmacists with other primary responsibilities [10]. Studies of community and critical access hospitals have described utilizing staff pharmacists and hospitalists as champions are unclear on workload and effort allocation [29–32]; one study described ASP interventions as burdensome to the pharmacist workflow [33]. To address these limitations, several pragmatic strategies have been explored to extend stewardship services to resource-limited settings: Telehealth-based programs have supported critical access hospitals with antimicrobial utilization without on-site ID specialists; regional collaboratives promote shared infrastructure, benchmarking, and peer-to-peer support among community hospitals; and collaboration with academic medical centers to establish system-wide ASPs can provide access to shared resources [33–35]. Regardless, given that most respondents reported themselves as an academic medical center, the survey results may not fully reflect perspectives from community or critical access hospitals. Inclusion of a greater proportion of community and critical access hospitals could have captured more resource-limited programs and modified variability in effort allocation and initiative performed.

Despite guidance on the multidisciplinary support for ASPs, this survey provides insight into the inconsistent effort allocation, especially for ancillary members such as informatics and data analysts. In addition, this survey also points out the lack of fee-for-service billing for ASPs and reporting of compensatory methods for participating members of on-call services. Given the goals of ASPs aligning with cost and harm reduction without generating net revenue, programs often have difficulty advocating for additional FTEs due to lack of direct-billing opportunities [36]. Although a deep dive into the financial landscape of ASPs is out of the scope of this manuscript, institutions should collaborate with administrators on pertinent financial outcomes and publish relevant findings [37].

For ASP initiatives performed after hours as part of on-call model, respondents indicated that major initiatives include answering adult ASP/ID questions, PRA, and answering microbiology inquiries. However, the proportion of respondents with an ASP participating in on-call for initiatives performed after hours was lower than during traditional working hours. Most respondents indicated that on-call staffing took place on weekends during daytime and major holidays, although 14 of 24 (58%) always had an ASP available. Our survey could not differentiate on initiatives performed during different time periods ASP was on-call; however, this may rationalize the reduced proportion of initiatives performed after hours to account for activities that may not immediately impact patient outcomes (eg, intravenous-to-oral transitions, review of high-risk antimicrobials for *C difficile* infection). Physicians and pharmacists participated in a similar number of shifts for on-call staffing, and a noteworthy proportion of respondents indicated that pharmacy residents also participated in ASP on-call. This promotes learner involvement with ASP initiatives, which has been previously demonstrated to be of benefit [18, 19].

This cross-sectional survey study has some important limitations. First, this was an electronic survey distributed by email to several ASP-related listservs to promote a high number of potential respondents; however, this limits an opportunity to establish a response rate to investigate whether potential respondents were not affiliated with ASPs or otherwise declined to participate. The latter scenario may be attributed to an extensive number of detailed questions, despite attempts to enhance feasibility of survey completion via pilot testing. In addition, while these listservs were inclusive of contacts outside of the US, responses were only received from US-based institutions. Second, characteristics derived from applicable questions with an “other” response may have been misclassified despite careful author review of comments provided by respondents rationalizing that choice. Third, this survey could not distinguish the level by which initiatives were performed: for example, one institution could have been performing PAF of all antimicrobials; meanwhile, another may have only performed PAF for broad-spectrum antibiotics such as carbapenems and fluoroquinolones. Similarly, the timing for when initiatives were performed after hours may have varied as previously mentioned. The additional number of questions required to specify these details would have increased survey length and potentially reduced the number of completed surveys. Last, this survey did not inquire about perceived workload or burnout associated with the reported ASP models, which may influence the feasibility of implementing a particular model and the range of initiatives performed.

## CONCLUSIONS

This survey serves as a reference for the institutional structure, initiatives, and working hours of ASPs based on on-call model participation. These findings provide context for participating members, effort allocation, and the types of initiatives taking place outside traditional working hours. Differences in effort assignment and initiatives performed between programs with and without on-call models may reflect on the prioritization of core ASP interventions and the delegation of certain initiatives to clinicians without formal ASP responsibilities.

## Supplementary Material

ofaf722_Supplementary_Data

## Supplementary Data

Supplementary materials are available at *Open Forum Infectious Diseases* online. Consisting of data provided by the authors to benefit the reader, the posted materials are not copyedited and are the sole responsibility of the authors, so questions or comments should be addressed to the corresponding author.
