# Supplementary material for: Beyond the 9 to 5: A Cross-sectional Survey of Adult Antimicrobial Stewardship Programs in the United States on Their Initiatives and Resources Based on On-call Model Participation
Source: Open Forum Infect Dis. 2025 Dec 15;12(12):ofaf722. doi: 10.1093/ofid/ofaf722 (PMC12702616; doi:10.1093/ofid/ofaf722)
Supplement: ofaf722_Supplementary_Data [file ofaf722_supplementary_data.docx]

**Antimicrobial Stewardship On-Call Models and Practices in the United States**

Collaborators: Krutika Hornback, Samantha Brace, Aaron Hamby, Rachel Burgoon, Zachary Gruss, Taylor Morrisette, Gustavo Alvira-Arill, Alexandra Mills, Richard Lueking, Stephen Thacker

Summary:

Antimicrobial stewardship programs (ASPs) are critical in improving patient care, reducing morbidity and mortality, and optimizing clinical outcomes by ensuring the appropriate use of antimicrobials.^1,2^ These programs aim to minimize the misuse and overuse of antibiotics, which are key factors in the development of antibiotic resistance – a significant threat to global health. Evidence has shown that ASPs can lead to better patient outcomes, including mortality rates, shorter hospital length of stay, and lower healthcare costs, all while improving the quality of care provided to patients.^1-3^ By promoting the judicious use of antimicrobials, ASPs help to prevent the emergence of multidrug-resistant organisms, reduce the incidence of healthcare-associated infections, and ensure that effective treatments remain available for the future.

To further validate the patient care benefits of ASPs, various accrediting and oversight agencies (CMS, The Joint commission, DNV) require their presence in inpatient and outpatient settings.^4-6^ However, there is paucity of guidance surrounding the optimal staffing, timings, responsibilities and compensation of ASPs beyond regular work hours (8a – 5p for the purposes of this project) or “ASP On-Call.” To optimize our local “ASP On-Call” program, we have recently completed a quality improvement project that has shown that institutions around the nation offer a wide range of staffing models (e.g., pharmacists only vs. pharmacists plus physicians, inclusion of weekends, etc.), services (e.g., preauthorization vs. prospective audit and feedback, sterile site culture review, etc.), and incentives (compensatory time off vs monetary compensation).

Survey:

This will be an electronic survey-based study among stewards and infectious diseases clinicians at institutions that utilize an on-call program across the United States. The primary aim is to provide structural guidance for institutions looking to implement and/or optimize their current “ASP On-Call” model. A 22-question survey using the REDCap® platform will be distributed to a targeted email group via various infectious diseases-based organizations listservs to identify institutions with an ASP On-call program (Infectious Diseases Society of America, Pediatric Infectious Diseases, Infectious Diseases educator Network, American College of Clinical Pharmacists Infectious Diseases PRN and SERGE-45). The survey will be distributed with a 4-week window for return. A reminder at 2 weeks will be sent. Pilot testing will be conducted to identify survey errors and estimated time to completion. All surveys with at least 33% of the items completed will be eligible for analysis. Descriptive statistics will be used to summarize the data.

Timeline and Deliverables:

| August 2024 | Survey tool developed; protocol finalized |
| --- | --- |
| September 2024 | Survey distributed |
| October 2024 | Survey data collated and analyzed |
| November 2024 | Abstract developed and submitted to ECCMID 2025 (April in Vienna, Austria) |
| Spring 2025 | Manuscript Developed |
| Summer 2025 | Manuscript submitted for targeted journal (e.g.,OFID, CID etc) |

References:

1. Barlam TF, Cosgrove SE, Abbo LM, et al. Implementing an Antibiotic Stewardship Program: Guidelines by the Infectious Diseases Society of America and the Society for Healthcare Epidemiology of America. Clin Infect Dis. 2016 May 15;62(10):e51-77. doi: 10.1093/cid/ciw118.

2. Dellit TH, Owens RC, McGowan JE Jr, et al. Infectious Diseases Society of America; Society for Healthcare Epidemiology of America. Infectious Diseases Society of America and the Society for Healthcare Epidemiology of America guidelines for developing an institutional program to enhance antimicrobial stewardship. Clin Infect Dis. 2007 Jan 15;44(2):159-77. doi: 10.1086/510393.

3. Davey P, Marwick CA, Scott CL, et al. Interventions to improve antibiotic prescribing practices for hospital inpatients. Cochrane Database Syst Rev. 2017 Feb 9;2(2):CD003543. doi: 10.1002/14651858.CD003543.pub4.

4. “New and Revised Requirements for Antibiotic Stewardship.” R3 - Requirement, Rationale, Reference, 2022, www.jointcommission.org/-/media/tjc/documents/standards/r3- reports/r3_antibioticstewardship_july2022_final.pdf. Accessed August 12, 2024.

5. Infection Prevention and Control and Antibiotic Stewardship Program Interpretive Guidance Update | CMS. https://www.cms.gov/medicareprovider-enrollment-andcertificationsurveycertificationgeninfopolicy-and-memos-states-and/infection-preventionand-control-and-antibiotic-stewardship-program-interpretive-guidance-update. Accessed August 12, 2024.

6. Healthcare advisory notices. DNV. https://www.dnv.us/supplychain/healthcare/advisory/
